# Model-based parametric study of frontostriatal abnormalities in schizophrenia patients

**DOI:** 10.1186/1471-244X-10-17

**Published:** 2010-02-27

**Authors:** Shoji Tanaka

**Affiliations:** 1 Department of Information and Communication Sciences, Sophia University, Tokyo 102-8554, Japan

## Abstract

**Background:**

Several studies have suggested that the activity of the prefrontal cortex (PFC) and the dopamine (DA) release in the striatum has an inverse relationship. One would attribute this relationship primarily to the circuitry comprised of the glutamatergic projection from the PFC to the striatum and the GABAergic projection from the striatum to the midbrain DA nucleus. However, this circuitry has not characterized satisfactorily yet, so that no quantitative analysis has ever been made on the activities of the PFC and the striatum and also the DA release in the striatum.

**Methods:**

In this study, a system dynamics model of the corticostriatal system with dopaminergic innervations is constructed to describe the relationships between the activities of the PFC and the striatum and the DA release in the striatum. By taking published receptor imaging data from schizophrenia patients and healthy subjects into this model, this article analyzes the effects of striatal D2 receptor activation on the balance of the activity and neurotransmission in the frontostriatal system of schizophrenic patients in comparison with healthy controls.

**Results:**

The model predicts that the suppressive effect by D2 receptors at the terminals of the glutamatergic afferents to the striatum from the PFC enhances the hypofrontality-induced elevation of striatal DA release by at most 83%. The occupancy-based estimation of the 'optimum' D2 receptor occupancy by antipsychotic drugs is 52%. This study further predicts that patients with lower PFC activity tend to have greater improvement of positive symptoms following antipsychotic medication.

**Conclusion:**

This model-based parametric study would be useful for system-level analysis of the brains with psychiatric diseases. It will be able to make reliable prediction of clinical outcome when sufficient data will be available.

## Background

Patients with schizophrenia show hypofrontality, which has been suggested to be responsible for cognitive impairment and negative symptoms [1-9]. Hypofrontality is observed even in prodromal stages of schizophrenia, indicating that hypofrontality itself is not sufficient for the onset of symptoms of schizophrenia but is a high-risk or vulnerability marker [10-12]. Therefore, hypofrontality would be a trait-like abnormality that underlies schizophrenia. On the other hand, hyperactivation of the dorsolateral prefrontal cortex (DLPFC) has often been observed during performing a working memory task [13-15]. The occurrence of hyperactivation of the DLPFC seems to be dependent on an intrinsic brain state as well as task performance and subjective efforts so that the observation has not necessarily been consistent or reproducible. A recent computational analysis of the DLPFC circuit dynamics suggests that the DLPFC circuit tends to be unstable under cortical hypodopaminergic conditions. This could lead to state-dependent variability of DLPFC activation that is considered to be much larger than mere individual differences [16]. Being consistent with this, schizophrenia patients showed much lower test-retest reliability than normal subjects in the working memory activation of the DLPFC, intraparietal sulcus, and insula [17]. It would, therefore, be possible to distinguish seemingly inconsistent hyperactivation of the DLPFC, a state-dependent phenomenon, from trait-like hypofrontality.

The activity of the PFC would have an influence on the activity of the striatum via the glutamatergic frontostriatal projection. Hypofrontality would then lead to a decrease in the activity of the striatal medium spiny neurons (MSNs). This would increase the DA concentration in the striatum due to the disinhibition of the DA neurons to which the MSNs send axons. As a consequence, the activity of the PFC and the DA level in the striatum would have a negative correlation, which is consistent with experimental observations [18-21]. Interestingly, the elevated DA levels in schizophrenic patients were associated with the improvement of positive symptoms by antipsychotic medication [22].

Many antipsychotic drugs have been targeting dopamine receptors, especially striatal D2 receptors [23]. Striatal MSNs have D1, D2 and other subtypes of DA receptors on the soma [24,25]. There are D2 receptors also at presynaptic terminals of the glutamatergic afferents from the PFC [26,27] as well as on the dopaminergic fibers [26-28]. High densities of D2 receptors in the striatum would enable DA to modulate powerfully the activity of the striatal neurons and information flow through the striatum [29]. It is suggested that DA gates the throughput of sensorimotor and incentive motivational inputs to the striatum and that DA is a "gatekeeper" for glutamate input to the striatum [30]. In schizophrenic patients, striatal DA turnover is elevated [31] and the baseline DA level in the striatum is increased [22]. The elevated levels of DA would overstimulate the D2 receptors in the striatum. The D2 receptors on the glutamatergic terminals would suppress the glutamatergic input to the striatum from the PFC, which may work as a DA filter of the input [26,27,32]. On the other hand, the roles and effects of the activation of DA receptors other than D2 receptors in the corticostriatal system remain less unambiguous. The aim of this article is to analyze how these D2 effects alter the characteristics of the frontostriatal system in patients with schizophrenia and healthy controls by using the receptor binding theory and a circuit model of the frontostriatal system. This article will also explore the possibility of the computational approach in psychiatric research.

## Methods

### Receptor binding

Binding potential (BP) of D2 receptors is given by [33](1)

The symbols used in the above equation and in the following equations are listed in Table 1.

**Table 1:** Symbols used in the model.

*a*	Coefficient that represents the presynaptic suppression of DA release by the D2 autoreceptor
*b*	Coefficient that represents the presynaptic depression of the frontostriatal glutamatergic neurotransmission by D2 receptor activation
*B*_max_	relative D2 receptor density
*BP*_AMPT_	Binding potential of the D2 receptor after DA depletion
[*DA*]	Extracellular concentration of endogenous DA
[*DA*]_*AMPT*_	Extracellular concentration of endogenous DA after depletion
*F*	Free synaptic concentration of the administered antipsychotic drug
*f *(*x*)	Activation function: *f *(*x*) = tanh (*x*), *x *≥ 0
*K*_*APD*_	Dissociation constant of the D2 receptor for the administered antipsychotic drug
*K*_*d*_	Dissociation constant of the D2 receptor for the radiotracer
*K*_*DA*_	Dissociation constant of the D2 receptors for endogenous DA
*P*	D2 receptor occupancy by endogenous DA
*P*_*APD*_	D2 receptor occupancy by the administered antipsychotic drug
*P*_*DA(APD)*_	D2 receptor occupancy by endogenous DA competing with the administered antipsychotic drug
*τ*_*d*_	Time constant of the DA neurons
*τ*_*s*_	Time constant of the striatal neurons
*τ*_*y*_	Time constant of DA release
*V*_*ps*_	Normalized connectivity coefficient of the frontostriatal projection
*W*_*dy*_	Coefficient representing DA releasability
*W*_*ps*_	Connectivity coefficient of the frontostriatal projection
*W*_*sd*_	Connectivity coefficient of the projection from the striatum to the DA nuclei
*x*_*d*_	Population activity of the midbrain DA nuclei
*x*_*p*_	Population activity of the PFC
*x*_*s*_	Population activity of the striatum
*y*	DA release in the striatum: *y *= [*DA*]

Drugs such as alpha-methyl-p-tyrosine (AMPT), a competitive and reversible inhibitor of tyrosine hydroxylase, reduce the extracellular concentration of endogenous DA significantly [22,34]. Comparing the BP before and after the administration of AMPT, we have(2)

for both schizophrenia patients and healthy controls. In the above equation, we assumed that the D2 receptor density, *B*_max_, did not change by acute administration of AMPT. This has been confirmed in rats [34] but not in humans. In schizophrenia patients, however, there is no reason that the receptor density, *B*_max_, is the same with that of healthy controls. Therefore, we distinguish the receptor density of schizophrenia patients (SZ) from that of healthy controls (HC) by using *B*_max, *SZ *_and *B*_max, *HC*_, respectively. Similarly, the extracellular DA concentration is different between patients and healthy controls, which are denoted by [*DA*]_*SZ *_and [*DA*]_*HC*_, respectively. The ratio of the BP of patients to that of healthy controls is given by(3)

for both before and after AMPT. Here the dissociate constant for DA, *K*_*DA*_, is also assumed to be unchanged in schizophrenia patients. The occupancy of the D2 receptor by DA is given by(4)

Using BP data obtained from receptor imaging studies, we are able to estimate the receptor occupancies for both schizophrenia patients and healthy controls.

### Circuit model

**Figure 1:**
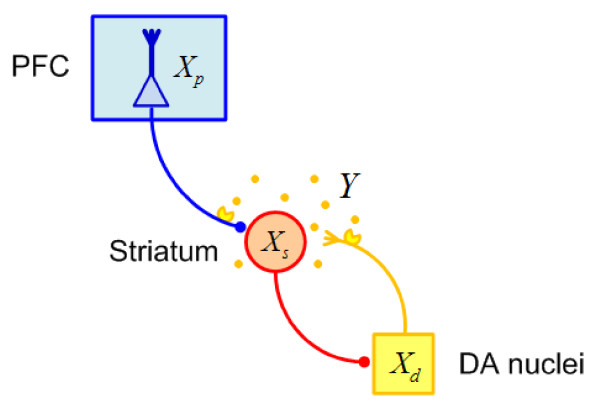
**Circuit diagram of the frontostriatal system**. The frontostriatal projection is glutamatergic (blue) and the axons from the striatal neurons to the midbrain DA neurons are GABAergic (red). D2 receptors are depicted at the terminals of the frontostriatal axon and the dopaminergic fiber in the striatum. Dots around the striatal neuron represent DA, which is released from the dopaminergic fiber (yellow).

The circuit model consists of the three brain regions; i.e., the PFC, the striatum, and the midbrain DA nuclei such as the substantia nigra and the ventral tegmental area (Fig. 1). It includes the effects of the stimulation of D2 receptors at the glutamatergic terminals of the frontostriatal projection and the autoreceptors on the dopaminergic terminals in the striatum. The dynamics of the population activities of the brain regions, the DA release in the striatum, and D2 receptor occupancy are given by(5)

The symbols used in the equations are also listed in Table 1.

In the equilibrium state (d/dt = 0), we have(6)

Using the linear approximation of the activation function *f*(*x*) ≈ *x *and introducing new variables: *X*_*p *_≡ *τ*_*s*_*τ*_*d*_*τ*_*y*_*W*_*ps*_*W*_*sd *_*W*_*dy *_*x*_*p*_/*K*_*DA*_, *X*_*s *_≡ *τ*_*d*_*τ*_*y*_*W*_*sd*_*W*_*dy *_*x*_*s*_/*K*_*DA*_, *X*_*d *_≡ *τ*_*y *_*W*_*dy *_*x*_*d*_/*K*_*DA*_, *J*_*d *_≡ *τ*_*y *_*W*_*dy *_*I*_*d*_/*K*_*DA*_, and *Y *≡ *y*/*K*_*DA*_, we rewrite the above equations as(7)

From these equations, we have the relationships between *X*_*p *_and *X*_*s *_as well as between *X*_*p *_and *Y *as(8)

or(9)

The variables have the physiological constraints that all of them are positive (*X*_*p*_, *X*_*s*_,* X*_*d*_, *Y *> 0)

## Results

### The frontostriatal system

**Figure 2:**
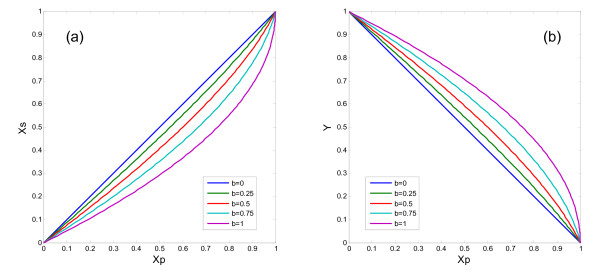
**Dependences of the striatal activity, *X*_*s*_, and the normalized DA release, *Y*, on PFC activity, *X*_*p*_**. The D2 effects become larger for higher values of *b*, the coefficient representing the presynaptic depression of the frontostriatal glutamatergic neurotransmission by D2 receptor activation.

Figure 2 shows the relationships between *X*_*p *_and *X*_*s *_as well as *X*_*p *_and *Y*, which are given by Eq. (8), with five different values of the D2 receptor activation coefficient (*b *= 0, 0.25, 0.5, 0.75, 1.0). We assumed *J*_*d *_= 1 for simplicity. The activation of the D2 receptor decreases *X*_*s *_and increases *Y*. As a result, the *X*_*s *_vs *X*_*p *_curve is convex downward and the *Y *vs *X*_*p *_curve is convex upward as shown in Fig. 2. The amount of the enhancement of the DA release is generally large; it is 0.55 (*b *= 1.0) compared to 0.30 (*b *= 0) when *X*_*p *_= 0.7. That is, for the modest activation of the PFC, the DA release increases by 83% when the coefficient of the D2 receptor stimulation increases from 0.0 to 1.0.

**Figure 3:**
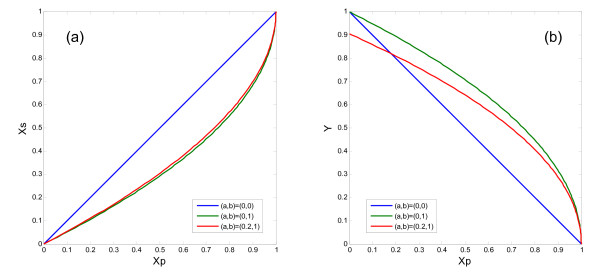
**Effects of the activation of the D2 receptors on the striatal activity, *X*_*s*_, and the normalized DA release, *Y***. The effect of the D2 autoreceptors on the dopaminergic fibers from the midbrain is specified by *a*. The effect of the D2 receptors on the glutamatergic terminals from PFC pyramidal neurons is specified by *b*.

The effects of autoreceptor activation, which are also given by Eq. (8), are depicted in Fig. 3. The effect on the activity of the striatum, *X*_*s*_, is small (Fig. 3a). The autoreceptor activation reduces the DA release, and this effect is larger when the activity of the PFC, *X*_*p*_, becomes lower (Fig. 3b). For example, the amount of DA release is 0.30 without D2 hetero- and autoreceptors, 0.55 with only the D2 heteroreceptor, and 0.50 with both the hetero- and autoreceptors (Table 2).

**Table 2:** Striatal population activity, *X*_*s*_, and DA release, *Y*, for different D2 effects (representing by the coefficients of *a *and *b*) when the PFC is modestly activated (*X*_*p *_= 0.7).

	a = 0		a = 0.2
	
	b = 0	b = 1	
*X*_*s*_	0.7	0.45	0.46
*Y*	0.3	0.55	0.5

### Dopamine depletion

The occupancy of D2 receptors in the striatum was estimated from the results of DA depletion studies [22,34-38]. The estimated occupancies are summarized in Table 3. There is only one study at present that gives the occupancy in both healthy subjects and schizophrenia patients [22]. The others studied either schizophrenia patients or healthy subjects. Because there is considerable variability in the occupancy among studies, we here set two models as follows.

**Table 3:** Striatal D2 receptor occupancies estimated from the receptor imaging studies.

	D2 occupancy		
	HC	SZ	References
	12%	21%	Abi-Dargham et al. (2000) [22]
	45%	-	Erlandsson et al. (2003) [35]
	26%	-	Laruelle et al. (1997) [34]
	13%	-	Riccardi et al. (2008) [36]
	22%	-	Verhoeff et al. (2001) [37]
	-	16%	Voruganti et al. (2001) [38]

average	23.6%	-	

There is only one study at present that gives the occupancy in both healthy subjects and schizophrenia patients.

#### Model 1

The first model comes from the study [22]. The D2 receptor occupancy is estimated to be 12% in healthy subjects and 21% in schizophrenia patients by assuming the DA depletion of 70%. The relative value of the D2 receptor density, *B*_max_, is also estimated to be 1.0 in the healthy subjects and 1.2 in the patients. In this case, the extracellular DA concentration normalized by the dissociation constant, *Y *= [*DA*]/*K*_*DA*_, is 0.136 in the healthy subjects and 0.266 in the patients.

#### Model 2

The second model comes from averaging of the results for healthy subjects because there are several different studies from healthy subjects. The averaged D2 occupancy is 23.6%. From the ratio of HC: SZ = 12%: 21% in Model 1, this extrapolates to the D2 receptor occupancy in patients of 23.6% × 21/12 = 41.3%. Model 2, therefore, uses the values of 24% and 41% for healthy subjects and patients, respectively. The relative values of are assumed to be the same with Model 1. In Model 2, the extracellular DA concentration normalized by the dissociation constant, *Y *= [*DA*]/*K*_*DA*_, is 0.316 in healthy subjects and 0.695 in schizophrenia patients. These results are summarized in Table 4.

**Table 4:** Normalized D2 receptor densities, D2 receptor occupancies by DA, and normalized extracellular DA concentrations in healthy subjects (HC) and schizophrenia patients (SZ) in Model 1 and Model 2.

	Model 1		Model 2	
	HC	SZ	HC	SZ
*B*_max_	1	1.2	1	1.2
*P*	12%	21%	24%	41%
[*DA*]/*K*_*DA*_	0.136	0.266	0.316	0.695

### Schizophrenia patients vs healthy subjects

In this section, we compare the dependences of the glutamatergic synaptic efficacy and the PFC activity on the D2 receptor activation between schizophrenia patients and healthy subjects. Because the results will be different between Model 1 and Model 2, we analyze them one by one. From Eq. (9), the normalized frontostriatal synaptic weight or the glutamatergic synaptic efficacy and the PFC activity are given by(10)

And we have the differences of *V*_*ps *_and between patients and healthy subjects as(11)

**Figure 4:**
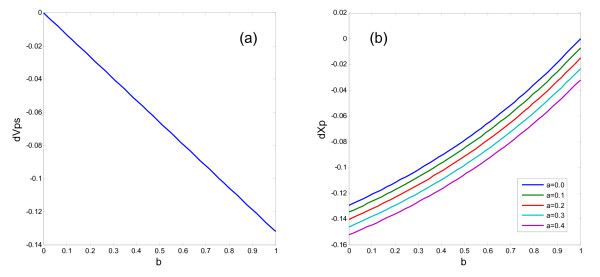
**Differences of the normalized glutamatergic synaptic efficacy and the PFC activity between schizophrenia patients and healthy subjects (i.e., those in SZ - those in HC), which are given by Eq. (11), in Model 1**.

**Figure 5:**
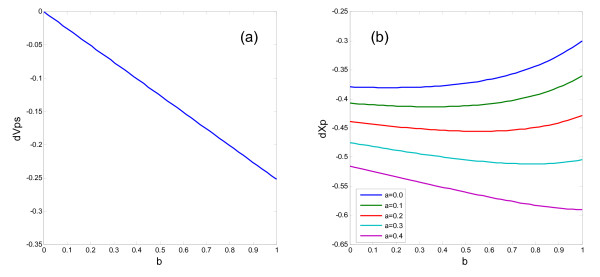
**Differences of the normalized glutamatergic synaptic efficacy and the PFC activity between schizophrenia patients and healthy subjects (i.e., those in SZ - those in HC), which are given by Eq. (11), in Model 2**.

Because *B*_max _= 1.0 (HC) vs 1.2 (SZ), the values of *a *and *b *in patients should be 1.2 times of those in healthy subjects. Both the differences given by Eq. (11) in Model 1 are depicted in Fig. 4. They are depicted against the D2 receptor activation coefficient, *b*, with different values of the autoreceptor activation coefficient, *a*. The difference in the normalized frontostriatal synaptic weight does not depend on the autoreceptor activation coefficient as shown in Eq. (11) (Fig. 4a). On the other hand, the PFC activity depends on the autoreceptor activation coefficient (Fig. 4b). Figure 5 shows the differences of the normalized frontostriatal synaptic weight and the PFC activity between schizophrenia patients and healthy subjects in Model 2.

### Optimum D2 receptor occupancy by antipsychotics

The administration of an antipsychotic drug causes competitive binding at D2 receptors. The occupancy of the D2 receptor by endogenous DA under the existence of an antipsychotic drug is given by(12)

The above equation shows that the occupancy of the D2 receptor by endogenous DA is generally reduced by the competitive binding with the antipsychotic drug. Similarly, the occupancy of the D2 receptor by the administered antipsychotic drug is given by(13)

We here assume that the optimum D2 antagonistic effect of antipsychotic drugs is expected when the *net binding *of endogenous DA to the D2 receptor in schizophrenia patients is reduced to that in healthy controls, that is(14)

or(15)

From Table 4, where P_*DA,HC *_is 12% in Model 1 and 24% in Model 2, the left hand side of the above equation is 10% in Model 1 and 20% in Model 2. Using Eqs. (12) and (13) as well as the values of [*DA*]/*K*_*DA *_in Table 4, we estimate the optimum free extracellular concentration of the antipsychotic drug divided by the dissociation constant and the occupancy of the D2 receptor by the administered antipsychotic drug as (*F*_*opt*_/*K*_*APD*_,P_*APD*_) = (1.39, 52.3%) in Model 1 and (1.78, 51.2%) in Model 2. The optimum antipsychotic effect requires the dose of an administrating drug that is higher in Model 2 than in Model 1. However, the occupancy of the D2 receptor by the administered drug is slightly lower in Model 2. This is because the DA release in Model 2 is higher than in Model 1. The total occupancy by DA and the drug, *P*_*DA(APD),SZ *_+ *P*_*APD*_, is also higher in Model 2, which is 71.2% versus 62.3% in Model 1.

### Prediction of clinical outcome

Abi-Dargham et al. [22] conducted 6-week antipsychotic medication of inpatients of schizophrenia (*n *= 14). The drugs used were olanzapine (*n *= 8), risperidone (*n *= 2), quetiapine (*n *= 2), clozapine (*n *= 1), and haloperidol (*n *= 1); benzodiazepine medications were added as needed. They measured AMPT-induced increases in D2 receptor BP before treatment and PANSS scores one and six weeks after treatment. The increase in D2 receptor BP induced by the acute administration of AMPT into the patients had a significant positive correlation with the improvement of positive symptoms after 6 weeks of the antipsychotic treatment (*R*^2 ^= 0.58, *p *= 0.0015). Changes in negative symptoms were not statistically significant. We have estimated the D2 receptor occupancy from the BP data. Our model describes the relationship between PFC activity and D2 receptor binding: Eq. (10) relates the PFC activity to the D2 receptor occupancy, which is given by Eq. (4). The extracellular DA concentration in Eq. (4) is obtained from Eq. (2) as(16a)

**Figure 6:**
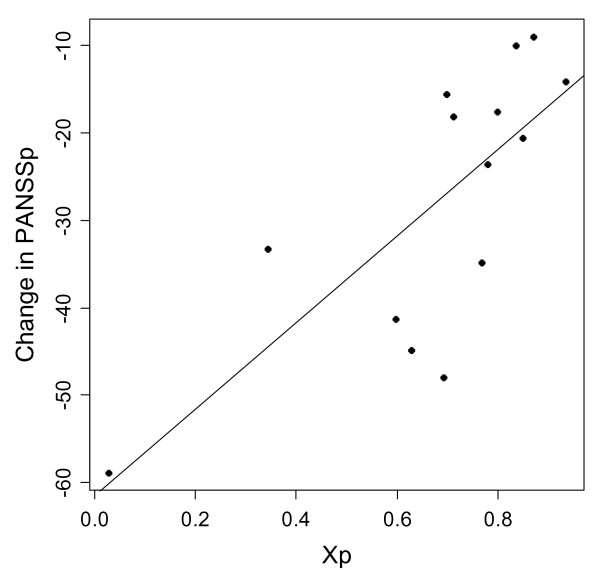
**Relationship between the change in the PANSS subscale for positive symptoms and the PFC activity, *X*_*p*_, when (*a, b*) = (0, 0)**. The regression line is Δ PANSSp = 49.6 *X*_*p *_- 61.5 (*R*^2 ^= 0.56, *p *= 0.002).

Using the assumption of *α *= 0.3 and the *β *values from Fig. 3 of [22], the improvement of positive symptoms can be associated with the PFC activity, as shown in Fig. 6. The regression model obtained from this transformed data set is Δ PANSSp = 49.6 *X*_*p *_- 61.5 (*R*^2 ^= 0.56, *p *= 0.002), where Δ PANSSp is the change in the PANSS subscale for positive symptoms. This model suggests that patients with lower PFC activity tend to have greater improvement of positive symptoms following subchronic (six weeks) antipsychotic treatment (note that Δ PANSSp = 49.6 *X*_*p *_- 61.5 < 0 for improvement).

## Discussion

The model in this article describes the inverse relationship between the PFC activity and the extracellular DA level in the striatum. This inverse relationship is accounted for by the circuitry of the frontostriatal system with dopaminergic innervation. The circuitry is consistent with anatomical study [39]. Functional neuroimaging studies [10,40] showing hypoactivation of both the PFC and the striatum during task performance, such as an oddball task and a working memory task, in schizophrenia patients also support this circuit model. Dysregulation of the frontostriatal system is responsible for the deficits in cognitive functions, including executive functions, in schizophrenia patients [41,42]. This article explored how much degree the circuit property of the frontostriatal system, which links the regional activities of the frontostriatal system and the striatal DA function, is relevant to schizophrenia.

The model describes that the stimulation of D2 receptors at the terminals of the frontostriatal projection enhances the striatal DA level due to the suppression of the glutamatergic input to GABAergic striatal neurons. With this model, the amount of elevation of the striatal DA level was estimated: When the PFC is modestly activated (*X*_*p *_= 0.7 in this model), the amount of DA elevation is maximally 83% when assuming *b *= 1.0 (i.e., 1.83 times larger compared with the case of no D2 receptor activation or *b *= 0). This elevation is reduced to 67% when D2 autoreceptors on the dopaminergic terminals are taken into account (assuming *a *= 0.2 in the model). The DA level might further be enhanced with increased proportions of the high-affinity states of D2 receptors, though this has not been taken into account in the model.

This study used receptor imaging data to estimate the amount of the DA release in the striatum in schizophrenia patients and healthy subjects. The result was then taken into the network model to calculate the glutamatergic input to the striatum and the population activity of the striatal neurons. It was thus possible to assume that the strength of D2 receptor activation is proportional to the D2 receptor occupancy by DA rather than the extracellular DA concentration. The combination of this model and the published receptor imaging data of schizophrenia patients and healthy controls enabled us to compare PFC activity that accounted for the alterations in the D2 receptor binding by endogenous DA in the striatum. The results suggest that the PFC population activity is reduced in schizophrenia patients, which is consistent with the hypofrontality hypothesis. Hypofrontality actually has unresolved issues; for example, on glutamatergic and GABAergic neurotransmission. In this article, however, the term is used to represent simply a reduction of the population activity of the PFC. The estimated amount of the reduction of the population activity depends on the D2 receptor occupancy by DA, which varies largely across the studies that have ever been made. Because the results of receptor imaging studies have a large variability, this article has chosen two model cases that could be representatives of the results. The reason that Model 2 led to a much larger difference in the PFC activity between patients and controls is that the experimentally estimated D2 receptor binding is higher than that in Model 1. Further accumulation of receptor imaging data would make this estimation more precise.

We have estimated the 'optimum' D2 receptor occupancy by an antipsychotic drug to be 52%. The estimation of the optimum occupancy of an antipsychotic drug is based on the assumption that the *net binding *of endogenous DA to the D2 receptors should be the same with that of healthy controls. Similar estimation was made previously [43], in which the authors made the assumption that the D2 receptor occupancy by DA should be the same with that of healthy controls and obtained the D2 receptor occupancy by an antipsychotic drug of 48%. This is slightly lower than our estimation because this estimation did not take the upregulation of D2 receptors into account. It is interesting to compare the estimated value of 52% with the measured occupancies of various antipsychotic drugs (Fig. 7 of [44]). The occupancies of many antipsychotic drugs are higher than this level and mostly in the range of 60-80%. The motor (extrapyramidal) side effects become prominent when the occupancy exceeds 75-80% [45]. Unlike other drugs, clozapine and quetiapine have the occupancies in the striatum that are mostly lower than 50%. The occupancy by quetiapine is lower than the occupancy by clozapine [46]. Therapeutic effects of these drugs appear to be achieved at the occupancy threshold that is lower than those of other antipsychotic drugs [46]. For other drugs, the occupancy should be lower than 75% to avoid extrapyramidal side effects. On the other hand, subjective well-being appeared to have a negative correlation with the striatal D2 receptor occupancy [47]. Therefore, the D2 receptor occupancy by many of the antipsychotic drugs should be in the range of 50-75% with a tradeoff between the efficacy and subjective experience. Agid et al. [23] examined the relationship between striatal and extrastriatal D2 occupancies by antipsychotic drugs and clinical effects, showing that striatal D2 occupancy predicted response in positive psychotic symptoms but not negative symptoms and the prediction by striatal D2 blockade was better than frontal, temporal, and thalamic occupancy. Their result (Fig. 1 of [23]) shows that striatal D2 blockade with the occupancy higher than 50% has a practical therapeutic effect on positive symptoms.

Schizophrenia patients with higher striatal D2 receptor occupancy tended to have greater improvement of positive symptoms after antipsychotic treatments [22]. If the hyperdopaminergic tone in the striatum is caused primarily by PFC hypoactivity, this would further indicate that hypofrontality could also be predictive of good response of positive symptoms to antipsychotic medication. This article examined this predictability of the model by using published data. The result shows that patients with lower PFC activity have larger improvements of positive symptoms as measured with PANSS positive scores. Hypofrontality has been generally associated with cognitive and negative symptoms in schizophrenia [1-9]. By linking hypofrontality to striatal hyperdopaminergic neurotransmission, the frontostriatal model proposed in this article has associated hypofrontality with the therapeutic effect on positive symptoms. This is testable by a combination of fMRI studies and clinical studies. Furthermore, a multivariate analysis of a data set from the combination of fMRI and PET receptor imaging studies will test the frontostriatal model more directly.

## Conclusion

This article described theoretically the relationship between regional activities in the frontostriatal system and striatal DA release. These quantities were compared between schizophrenia patients and healthy subjects by using the results from receptor binding studies. The results are consistent with hypofrontality. This study also predicts that patients with lower PFC activity tend to have greater improvement of positive symptoms following antipsychotic medication. The 'optimum' D2 receptor occupancy by antipsychotic drugs was estimated to be 52%.

## Competing interests

The author declares that he has competing interests.

## Authors' contributions

ST is the sole author of this study. He designed the study, directed the modeling and analysis and wrote the paper.

## Pre-publication history

The pre-publication history for this paper can be accessed here:

http://www.biomedcentral.com/1471-244X/10/17/prepub
