# Correction: Model-based parametric study of frontostriatal abnormalities in schizophrenia patients

**DOI:** 10.1186/1471-244X-10-93

**Published:** 2010-11-19

**Authors:** Shoji Tanaka

**Affiliations:** 1 Department of Information and Communication Sciences, Sophia University, Tokyo 102-8554, Japan

## 

After the publication of this work [1], the author became aware that it contains inaccurate descriptions as well as typographical errors. They should be corrected in order that the descriptions in the text and the results are consistent. The corrections are listed below with reasons. These corrections are superficial and do not affect the results and conclusions. The author regrets any inconvenience that these inaccuracies might have caused.

1. Page 3: Typographical errors: *γ *(gamma) in Eqs. (5) and (6) should be *γ*.

2. Page 4: In the description of Model 1, "the DA depletion of 70%" should be "the DA depletion of 75%" because the author used the value of 75% in the calculation.

3. Page 4: The description of Model 2 contains unnecessary sentences: "The averaged D2 occupancy is 23.6%, From the ratio of ...", and the whole description should be replaced by

### Model 2

The DA occupancy of 12% in healthy subjects is the lowest among the studies summarized in Table three. Model 2 assumes higher values of D2 occupancies, which are 24% for healthy subjects and 41% for schizophrenia patients. This assumption gives the extracellular DA concentration normalized by the dissociation constant,*Y *= [*DA*]/*K_DA _*, of 0.316 in healthy subjects and 0.695 in patients, respectively (Table four).

4. Page 5: Table three (Table 1 in this manuscript) contains an erroneous citation of the paper: Erlandsson et al. (2003), which provides D2 receptor occupancy by risperidone rather than by dopamine. The D2 receptor occupancy in patients from the study by Voruganti et al. (2001) is also erroneously written, which is 16%. The correct value is 39%. The average value of the occupancy in healthy controls is not used in the calculation in this study, so that it is to be omitted. Because the estimation of the occupancy was based on DA depletion rate, the value is to be specified in each cited study. Radiotracers and imaging techniques used in the cited studies are also specified for clarity. Taking all of these corrections, Table three should be replaced by

**Table 1:** Striatal D2 receptor occupancies by DA estimated from receptor imaging studies.

DA depletion	D2 occupancy		Radiotracer	Study
	HC	SZ		
0.25	12%	21%	[^123^I]IBZM	SPECT (Abi-Dargham *et al. *2000) [22]
0.20	26%	-	[^123^I]IBZM	SPECT (Laruelle *et al. *1997) [34]
0.29	13%	-	[^18^F]fallypride	PET (Riccardi *et al. *2008) [36]
0.29	22%	-	[^11^C]raclopride	PET (Verhoeff *et al. *2001) [37]
0.30	-	39%	[^123^I]IBZM	SPECT (Voruganti *et al. *2001) [38]

The value of DA depletion of 0.25, for example, corresponds to the depletion of 75% of endogenous DA.

5. Page 6: In Eqs. (12) and (13), *Y *should be replaced by [*DA*], so that the correct equations are

(12) $$P_{DA(APD)}=\frac{\frac{\left[DA\right]}{K_{DA}}}{1+\frac{\left[DA\right]}{K_{DA}}+\frac{F}{K_{APD}}}$$

(13) $$P_{APD}=\frac{\frac{F}{K_{APD}}}{1+\frac{\left[DA\right]}{K_{DA}}+\frac{F}{K_{APD}}}$$

6. Page 7: "Δ PANSS is the change" should be "Δ PANSS is the percent change".

7. Page 10: Reference 35 should be removed from the list because of the reason provided in Correction #4.

## Pre-publication history

The pre-publication history for this paper can be accessed here:

http://www.biomedcentral.com/1471-244X/10/93/prepub
